# How does community of inquiry matter? Mediating effects and configurational pathways linking project-based learning design to learning engagement

**DOI:** 10.3389/fpsyg.2026.1976588

**Published:** 2026-10-23

**Authors:** Tongfeng Xu, Peijun Qin, Siyuan Ma

**Affiliations:** School of New Media, Beijing Institute of Graphic Communication, Beijing, China

**Keywords:** animation education, community of inquiry, generative artificial intelligence, learning engagement, project-based learning

## Abstract

The widespread adoption of generative artificial intelligence (AIGC) has reshaped animation students’ skill profiles and the conditions of instructional design in project-based learning (PBL) studios. We examine whether and how perceptions of PBL design are associated with Learning Engagement (LE), treating AIGC-assisted PBL studios as the research context rather than testing generative AI as a model variable. Drawing on Community of Inquiry (CoI) theory, we examine Problem Driven (PD), Project Design (PDe), Project Implementation (PI), and Project Evaluation (PE) as antecedents; Cognitive Presence (CP), Social Presence (SP), and Teaching Presence (TP) as statistical mediators; and LE as the outcome. We analyzed two-wave survey data from 490 upper-year animation students using PLS-SEM and fsQCA. PBL and CoI were measured concurrently at T1, and LE at T2; indirect effects therefore represent indirect statistical associations rather than mediation with a fully established temporal sequence. PD, PDe, and PI showed significant positive direct associations with LE. PE’s zero-order and single-predictor associations were positive, but its direct path coefficient became significantly negative after controlling for the other PBL dimensions and CoI mediators. The coefficient was sensitive to model specification, suggesting possible statistical suppression; it should not be interpreted as evidence that evaluation itself inherently inhibits engagement. The three CoI dimensions showed significant statistical mediation associations across all 12 indirect paths. TP and SP had relatively higher point estimates of indirect statistical associations, but differences between indirect effects were not formally tested; no dimension is claimed to be optimal. PE’s positive indirect associations through CoI partially offset its negative net direct association without reversing the total association after adjustment. The extent of this offset may depend on the degree to which CoI-related experiences are concurrently reported. fsQCA identified seven high-engagement configurations. No individual PBL dimension was indispensable. CoI dimensions appeared as core or peripheral conditions, although not every configuration included all three. These findings clarify the dual pathways linking evaluation and engagement through indirect statistical associations and configurational conditions, and provide guidance for optimizing PBL under different resource constraints. Conclusions are limited to AIGC-assisted PBL studios and do not establish the instructional effects of generative AI.

## 1 Introduction

Animation education is at a critical juncture: it’s central instructional activities—conceptualizing and producing visual solutions—are among the tasks most susceptible to AIGC automation. Tools such as Midjourney and Stable Diffusion can now complete in minutes work that traditionally required weeks (Wang et al., 2024). As the production skills on which animation training has long relied rapidly lose their relative value, developing higher-order capabilities, including problem solving, critical reflection, and aesthetic judgment, has become increasingly urgent (Zhang and Lin, 2026). Technological support, however, does not automatically simplify learning. When students can generate visual solutions rapidly with AI, they face a fundamental tension between efficiency and critical judgment: they must use AI to extend their creative capabilities while independently evaluating its outputs (Lee, 2026). Helping students navigate this tension through instructional design is an immediate challenge for animation education. Among available pedagogical approaches, PBL has long been regarded as an effective strategy for developing complex capabilities because it emphasizes authenticity, autonomy, and collaboration (Mergendoller et al., 2013). Educational technology research has also documented the overall benefits of PBL for learning outcomes (Lara-Bercial et al., 2024). Yet targeted empirical evidence remains limited on whether its individual design dimensions are equally effective in fostering deep engagement in AI-enhanced studios. As technology reconfigures the skills underpinning animation practice, this study asks which specific PBL design dimensions can still foster students’ learning engagement, through what mechanisms, and under which conditions.

PBL is commonly operationalized along four dimensions: Problem Driven (PD), Project Design (PDe), Project Implementation (PI), and Project Evaluation (PE). Prior research using learning motivation as the outcome found that PD was a strong predictor, whereas PE had almost no direct predictive effect (Zhang and Lin, 2026). This apparent failure of evaluation provides the theoretical starting point for the present study. However, that finding established only the direction of the effects; it did not test why evaluation was ineffective or under what conditions its contribution might improve. We address this gap by examining the boundary conditions of evaluation effects. Both the earlier research and the present study are situated in educational settings experiencing rapid AIGC adoption, but GenAI itself is not an independent variable examined here.

Reporting differences in effects across design dimensions leaves a more fundamental question unanswered: through what mechanisms do PBL design features translate into learning outcomes? We therefore introduce Community of Inquiry (CoI) theory as a mediation framework. CoI conceptualizes meaningful learning experiences in terms of Cognitive Presence (CP), Social Presence (SP), and Teaching Presence (TP) (Garrison et al., 2001). We propose that the four PBL dimensions contribute to Learning Engagement (LE) indirectly by activating students’ CoI experiences in the studio—the cognitive challenges, social connections, and instructional support they perceive—rather than generating engagement solely through direct pathways. AIGC-assisted PBL studios provide a particularly informative setting for observing these indirect associations; AI itself is not the variable this study seeks to explain. In this setting, AI performs many production tasks, potentially differentiating the strength and form of the associations between PBL dimensions and CoI experiences. This differentiation is the focus of our inquiry. Whether the type, intensity, quality, or degree of dependence on AI use further moderates these relationships was not measured and remains a question for future research.

On this basis, we address three research questions: (1) Do the direct effects of individual PBL design dimensions on LE differ significantly, and does PE reproduce the pattern of ineffective evaluation reported previously? (2) Do the three CoI dimensions significantly mediate the relationships between PBL dimensions and LE, and how do their relative mediation strengths compare? (3) Are there multiple equifinal PBL-CoI configurations associated with high LE? These questions correspond, respectively, to tests of direct effects, tests of mediation mechanisms, and exploration of configurational pathways. The second question, particularly whether the indirect effects of PE through CoI can offset a potentially negative direct effect, is the study’s central theoretical concern. Generative AI (GenAI) is treated as a contextual feature and a sample eligibility criterion, not as an explanatory variable tested in the model. All participants attended courses using AIGC visual generation tools to support PBL studio instruction. However, we neither measured AI-related variables nor included a comparison group with no or low AI use. The study can therefore establish statistical associations and configurational conditions linking perceptions of PBL design, CoI experiences, and Learning Engagement, but cannot determine whether or how GenAI changes these relationships. All references to AIGC describe the research context rather than support inferences about AI effects. The conclusions apply only to this AIGC-assisted PBL context and cannot be attributed to GenAI itself.

## 2 Research model and hypotheses

### 2.1 Community of Inquiry

The central premise of CoI is that meaningful learning experiences emerge through the dynamic interplay of cognitive presence, social presence, and teaching presence (Garrison et al., 2001). Cognitive Presence refers to the extent to which learners construct and confirm meaning through sustained reflection and discourse. Social Presence denotes learners’ capacity to present themselves socially and emotionally within a community. Teaching Presence encompasses the design, facilitation, and direction of cognitive and social processes to support meaningful learning (Garrison et al., 2010). Grounded in social constructivism, this framework views learning as an integrated process of reflection, collaboration, and guided inquiry rather than a one-way transmission of information.

Since its introduction, the scope of the CoI framework has been progressively extended and tested. Early studies focused on fully online discussions; subsequent work extended the framework to blended learning, technology-enhanced face-to-face classrooms, and mobile learning environments. The literature broadly agrees that the three-dimensional structure remains robust across learning modalities, although the relative contributions of the dimensions and their patterns of interaction vary by context (Martin et al., 2022). Applying CoI to a new technological setting thus requires clarifying how that setting reshapes the relative contributions and pathways of the three presences, beyond re-establishing the framework’s structural validity.

These questions are particularly salient in AI-assisted PBL studios. AIGC changes the division of cognitive labor: AI generates substantial amounts of visual material, while students’ central cognitive tasks increasingly involve judgment and integration (Liu and Zhu, 2025). This shift may alter both the conditions that give rise to the three presences and their functions. Teaching Presence shifts from knowledge transmission toward the design of cognitive scaffolds, with instructors guiding students in selecting and integrating AI outputs. The interaction structure of Social Presence expands from interpersonal collaboration to human–AI collaboration, as AI-generated proposals become shared cognitive artifacts for team negotiation. The conditions that trigger Cognitive Presence also change: as AI accelerates production, opportunities for deep reflection may be compressed, making deliberately scheduled reflection more important for activating Cognitive Presence (Stenbom et al., 2026). These considerations motivate our analytical approach. We position CoI as a set of statistical mediators and configurational conditions linking PBL design to Learning Engagement, examining the relative contributions of the three presences and their combinations under different PBL design conditions. This moves CoI beyond a descriptive framework toward an explanatory analytical framework that can help account for why the same PBL design element is associated with different outcomes under different conditions. The discussion of AI and the division of cognitive labor establishes the theoretical premise for possible changes in the relative contributions of the three presences; it does not imply that AI is included as a model variable.

### 2.2 PBL dimensions and learning engagement

The construct of learning engagement refers to the psychological energy and behavioral effort students invest in learning activities (Fredericks et al., 2004; Panigrahi et al., 2021). It is closely related to, but analytically distinct from, learning motivation: motivation concerns why students learn, whereas engagement concerns how they participate in learning (Wang and Eccles, 2013). This distinction establishes the construct boundaries for positioning CoI as a mediating mechanism linking PBL design to Learning Engagement.

Empirical research supports the overall contribution of PBL to Learning Engagement (Almulla, 2020; Rehman et al., 2024), including deeper engagement than that observed under conventional instruction (Almulla, 2020). Most studies, however, examine PBL as an overall pedagogical approach, with limited attention to the differentiated contributions of individual design dimensions. This limitation is especially consequential in technology-enhanced settings, where AI tools may change the distribution of cognitive demands across PBL activities and, consequently, the relative importance of its dimensions. Although prior research examined learning motivation as the dependent variable, it identified a differentiated pattern: Problem Driven had the strongest effects on intrinsic motivational dimensions such as perceived challenge and enthusiasm; Project Design and Project Implementation had selective effects; and Project Evaluation had almost no direct effect (Zhang and Lin, 2026). Because motivation is an immediate antecedent of engagement (Eltahir and Alsalhi, 2025), this pattern suggests that PBL dimensions may also contribute differently to Learning Engagement. We pursue a further question: if Project Evaluation has no positive direct association with engagement, or even a negative one, does this imply that evaluation lacks value? Alternatively, might its value be realized indirectly through specific mechanisms, such as deep reflection (Cognitive Presence), peer dialogue (Social Presence), and instructor guidance (Teaching Presence)? This question is central to the study’s theoretical contribution. We therefore propose the following hypotheses:

*H1a*: Problem Driven has a significant positive effect on Learning Engagement.
*H1b*: Project Design has a significant positive effect on Learning Engagement.
*H1c*: Project Implementation has a significant positive effect on Learning Engagement.
*H1d*: The direct association between Project Evaluation and Learning Engagement is non-positive, whereas its indirect statistical associations through the three CoI dimensions are expected to be positive. The direction of the overall association depends on the relative strengths of the direct and indirect statistical associations.

It should be noted that the “direct association” in H1d refers to the net direct path coefficient in the structural model after controlling for the other PBL dimensions. It is not equivalent to the zero-order association or the coefficient when PE alone predicts LE. The zero-order association and the results for PE as the sole predictor are reported in the Results section.

### 2.3 Mediating roles of the three community of inquiry dimensions

We position the CoI framework as a mediating mechanism linking PBL design to Learning Engagement. Educational technology research has provided initial support for this logic: the three CoI dimensions significantly mediated the relationship between intelligent learning analytics technology and Learning Engagement, with the indirect effects through Teaching Presence, Cognitive Presence, and Social Presence jointly accounting for 57.1% of the total effect (Dong et al., 2026). We extend this logic from technological resources to instructional design, testing whether the four PBL dimensions are indirectly related to Learning Engagement through the three CoI dimensions.

In PBL studios, the proposed progression from design to learning experiences and then to engagement has three theoretically grounded pathways. First, Cognitive Presence may develop through visual argumentation activities in which students compare AI-generated and independently developed proposals. Such activities can counteract the compression of reflection time associated with technological acceleration (Zhang and Lin, 2026). Reframing AI outputs as cognitive objects to be evaluated, rather than final answers, can initiate sustained reflective meaning-making that translates into cognitive and behavioral engagement (Dong et al., 2026; Zawacki-Richter et al., 2019). Second, Social Presence may be supported when AI-generated discussion guidance provides structured prompts that scaffold dialogue, reduce disorder and stalled exchanges, and make group interaction feel manageable. Learners may then recognize that their group is engaging in substantive, productive social interaction, strengthening their sense of belonging (Zhai and Nehm, 2023). This process can cultivate emotional connections and shared responsibility for learning tasks, providing a foundation for an effective learning environment (Anderson et al., 2025). Third, Teaching Presence may arise through instructors’ Project Design and learning guidance, particularly explicit rules for AI use and criteria for critically selecting outputs. Such guidance helps students perceive clear learning directions and available support, strengthening trust and sustained engagement (Zhang and Zhang, 2024). In AI-enabled learning environments, the central instructional function increasingly shifts from knowledge transmission to cognitive scaffold design (Hassan, 2026), including teaching students how to collaborate with AI and assess the accuracy of its answers. This shift may further strengthen the robustness of the mediating mechanism. We therefore propose:

*H2a*: Cognitive Presence mediates the relationships between PBL dimensions and Learning Engagement.
*H2b*: Social Presence mediates the relationships between PBL dimensions and Learning Engagement.
*H2c*: Teaching Presence mediates the relationships between PBL dimensions and Learning Engagement.

### 2.4 Proposed theoretical model

The proposed theoretical model is presented in Figure 1.

**Figure 1:**
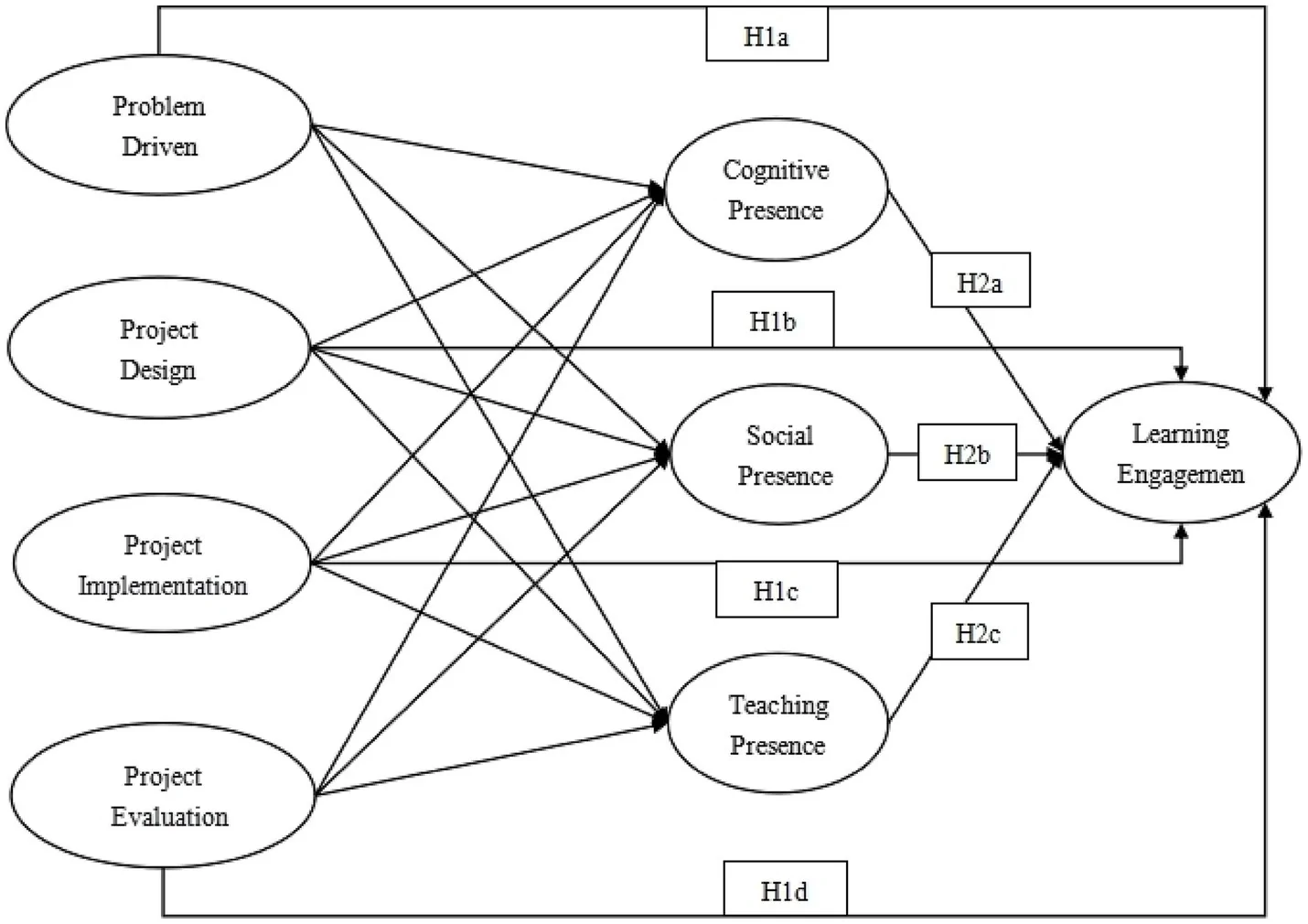
Proposed theoretical model.

## 3 Research methods

### 3.1 Research design and data collection

We adopted a quantitative design and a multistage questionnaire procedure to examine the proposed theoretical model. The model conceptualizes the four PBL dimensions (Problem Driven, Project Design, Project Implementation, and Project Evaluation) as antecedents, the three CoI dimensions (Cognitive Presence, Social Presence, and Teaching Presence) as mediators, and Learning Engagement as the outcome. The research proceeded in four stages: (1) developing a conceptual model and structural hypotheses based on CoI theory; (2) designing and pilot-testing the questionnaire (*n* = 30), with expert review and field feedback informing assessments of validity and reliability; (3) administering a two-wave time-lagged survey to reduce common method bias; and (4) analyzing the quantitative data using PLS-SEM and fsQCA to identify structural paths and configurational patterns.

The survey used a two-wave time-lagged design. At the first wave (T1), students reported their perceptions of the four PBL dimensions (Problem Driven, Project Design, Project Implementation, and Project Evaluation) and the three CoI dimensions (Cognitive Presence, Social Presence, and Teaching Presence). At the second wave (T2), we measured Learning Engagement. The waves were separated by 3 weeks to reduce consistency bias and strengthen the temporal ordering of the measurements (Widaman et al., 2010). This interval was intended to reduce immediate recall of the T1 questionnaire while limiting substantive changes in perceptions of the PBL course experience. Data were collected between March and June 2026 through the online Wenjuanxing platform. Participants were third- and fourth-year animation students at application-oriented undergraduate institutions. This population was selected for two reasons: students in earlier years had not yet systematically participated in PBL courses, whereas upper-year students had completed PBL studio courses and had course experience using AIGC tools to complete integrated projects, giving them basic experience of the core phenomenon examined in this study (Zhang and Lin, 2026).

At T1, 600 questionnaires were distributed and 550 were returned. At T2, 550 questionnaires were distributed, yielding 490 successfully matched responses. The final analytical sample comprised 490 students, corresponding to a T1–T2 matching rate of 89.09% and an attrition rate of 10.91%. Attrition primarily reflected nonparticipation at T2 (35 students), unmatched records (15 students), and incomplete information (10 students). All four PBL dimensions and all three CoI dimensions were measured concurrently at T1, whereas LE was measured at T2. The time-lagged design may reduce some common method bias and places the outcome after the T1 variables, but it does not establish that changes in PBL precede changes in CoI. Accordingly, the indirect effects estimated by PLS-SEM should be interpreted as indirect statistical associations linking T1 perceptions of PBL and CoI to T2 learning engagement, rather than mediation with a fully established temporal sequence. Testing the temporal mediation chain PBL → CoI → LE would require a three-wave longitudinal design measuring PBL design at T1, CoI experiences at T2, and LE at T3.

We used stratified convenience sampling, with institution type (comprehensive universities and art and design institutions) defining the strata to ensure representation of both categories. These institutions differ systematically in curriculum structure, staffing, and studio resources. Including both types helps capture the diversity of animation education and avoids restricting the sample to a single institutional model. Within each institution, quotas were allocated by year of study (third and fourth year) to ensure balanced representation. Each institution contributed approximately 45–50 students. The final sample of 490 was sufficient for structural equation modeling of moderate complexity (Hair et al., 2019). Participants were eligible if they: (1) attended a course or project that used AIGC visual generation tools to support PBL studio instruction during the 2025–2026 academic year; (2) were 19–26 years old; and (3) provided informed consent and participated voluntarily. The survey was promoted through course WeChat groups, class representatives, and academic advisers. A prize draw was used to encourage participation. Table 1 presents respondents’ demographic characteristics.

**Table 1:** Demographic characteristics of the respondents.

Variable	Category	Frequency	Percentage (%)
Gender	Male	221	45.1
	Female	269	54.9
Year of study	Third year	251	51.2
	Fourth year	239	48.8
Field of study	Animation design	196	40
	Digital media art	168	34.3
	Game design	126	25.7
Frequency of AI tool use	<1 time/week	88	18
	1–2 times/week	235	48
	≥3 times/week	167	34
Experience using AI tools	<6 months	52	10.6
	6–12 months	186	38
	>12 months	252	51.4

### 3.2 Questionnaire design and measurement

The measures were adapted from established scales widely used in the international literature, taking into account the characteristics of AIGC and the context of online learning in Chinese higher education. Adaptation involved five steps. First, two bilingual researchers independently translated the English scales into Chinese, and a third researcher back-translated them. The back-translated and original versions were compared for semantic equivalence. Second, five experts in animation education, educational technology, and psychometrics (two, two, and one, respectively) reviewed the items for content validity, clarity, and contextual appropriateness. Third, a pilot study with 30 students assessed item comprehension, completion time, and participant feedback. Fourth, wording was refined based on the expert reviews and pilot results. Finally, the questionnaire used a seven-point Likert scale ranging from 1 (strongly disagree) to 7 (strongly agree). Supplementary Appendix A1 provides the sources, adaptation rationale, and final items for each construct.

The four PBL dimensions were measured using a framework for analyzing PBL characteristics (Pan et al., 2021), with reference to an animation-specific PBL scale validated in animation education (Zhang and Lin, 2026). The scale comprised 25 items: six each for Problem Driven, Project Design, and Project Implementation, and seven for Project Evaluation. The three CoI dimensions were measured using an abbreviated version of the established Community of Inquiry instrument (Arbaugh et al., 2008), comprising five items each for Cognitive Presence, Social Presence, and Teaching Presence, for a total of 15 items. These measures were contextually adapted to AI-enhanced PBL studios. Learning Engagement was measured based on the framework of Jung and Lee (2018), covering three theoretical dimensions—behavioral, emotional, and cognitive engagement—with three items per dimension, for a total of nine items. In the measurement model, the nine items were modeled as direct indicators of a single reflective Learning Engagement construct, rather than as three first-order factors or a higher-order construct. This specification reflects the study’s focus on overall learning engagement rather than differences between dimensions. It does not negate the theoretical multidimensionality of LE; structural differences at the dimension level remain to be examined in future research.

### 3.3 Analytical techniques

We combined PLS-SEM and fsQCA to examine structural pathways and patterns of interaction. This dual analytical approach was intended to deepen the theoretical analysis and strengthen the robustness of the conclusions.

Structural equation modeling was conducted in SmartPLS 4.0, which is suitable for moderate sample sizes and non-normal data. The analysis included: (1) measurement model assessment covering reliability (Cronbach’s α and CR), convergent validity (AVE), and discriminant validity (Fornell-Larcker and HTMT criteria). The nine learning engagement items were included in the measurement model as direct indicators of a single reflective LE construct. We did not test a three-dimensional first-order structure or a higher-order structure for LE and therefore make no inferences about its measurement structure at the dimension level; (2) structural path analysis covering path significance (Bootstrapping with 5,000 resamples), explanatory power (*R*^2^), predictive relevance (*Q*^2^), and effect sizes (*f*^2^); and (3) analysis of indirect statistical associations. Bootstrapping with 5,000 resamples was used to test the indirect statistical associations linking each PBL dimension to LE through the three CoI dimensions. Because PBL and CoI were measured concurrently at T1, this analysis identifies patterns of statistical association linking T1 perceptions of PBL and CoI to T2 engagement; it does not establish the temporal sequence PBL → CoI → L E.

We used fsQCA 3.0 to identify multiple configurations associated with high LE, such as combinations of high or low perceived PBL and CoI. The procedure involved variable calibration, necessity analysis, truth table construction, identification of core and peripheral conditions, and presentation of the resulting configurations.

## 4 Results

### 4.1 Study 1 structural equation modeling (SEM)

We assessed the measurement model in SmartPLS 4.0, examining reliability, convergent validity, and discriminant validity.

#### 4.1.1 Reliability and convergent validity

As shown in Table 2, Cronbach’s *α* ranged from 0.791 to 0.942 across latent variables, and composite reliability (rho_c) ranged from 0.854 to 0.951. All values exceeded the recommended threshold of 0.700 (Hair et al., 2019), indicating satisfactory internal consistency. Average variance extracted (AVE) ranged from 0.504 to 0.682. Project Implementation had the lowest AVE (0.504), but still met the minimum criterion of exceeding 0.500 (Fornell and Larcker, 1981), supporting convergent validity.

**Table 2:** Measurement model: all items as direct indicators of their respective constructs.

Variables	Items	Factor loading	Cronbach’s alpha	rho_c	AVE
Problem Driven (PD)	PD1 PD2 PD3 PD4 PD5 PD6	0.755 0.749 0.768 0.738 0.756 0.628	0.840	0.875	0.539
Project Design (PDe)	PDe1 PDe2 PDe3 PDe4 PDe5 PDe6	0.839 0.680 0.686 0.687 0.839 0.637	0.838	0.873	0.536
Project Evaluation(PE)	PE1 PE2 PE3 PE4 PE5 PE6 PE7	0.752 0.770 0.752 0.675 0.770 0.571 0.700	0.856	0.879	0.513
Project Implementation (PI)	PI1 PI2 PI3 PI4 PI5 PI6	0.822 0.646 0.664 0.822 0.674 0.597	0.811	0.857	0.504
Cognitive Presence (CP)	CP1 CP2 CP3 CP4 CP5	0.803 0.758 0.778 0.810 0.686	0.830	0.878	0.590
Social Presence (SP)	SP1 SP2 SP3 SP4 SP5	0.735 0.777 0.753 0.681 0.719	0.791	0.854	0.539
Teaching Presence (TP)	TP1 TP2 TP3 TP4 TP5	0.743 0.735 0.721 0.824 0.680	0.805	0.859	0.551
Learning Engagement (LE)	LE1 LE2 LE3 LE4 LE5 LE6 LE7 LE8 LE9	0.838 0.834 0.838 0.821 0.823 0.824 0.820 0.811 0.822	0.942	0.951	0.682

The nine Learning Engagement items were included in the model as direct indicators of a single reflective LE construct. Behavioral, emotional, and cognitive engagement were not specified as three first-order factors, nor was a higher-order LE construct specified.

#### 4.1.2 Discriminant validity

We assessed discriminant validity using the Fornell and Larcker (1981) criterion. As shown in Table 3, the square roots of AVE for LE, CP, PD, PI, PDe, and PE exceeded their correlations with the other constructs. However, the correlation between TP and SP was 0.751, exceeding the square roots of AVE for both TP (0.742) and SP (0.734). Thus, the Fornell-Larcker criterion was not met for TP and SP. All HTMT values were below the conservative threshold of 0.85 (see Table 4), with a value of 0.801 for TP and SP, indicating no serious violations of discriminant validity. Taken together, the evidence supports discriminant validity for most constructs, but provides only partial support for the discriminant validity of TP and SP. Accordingly, we do not claim that discriminant validity has been fully established for all constructs. Paths involving TP and SP are interpreted cautiously, and this issue is discussed further in the limitations.

**Table 3:** Discriminant validity.

Variable	LE	TP	SP	CP	PD	PI	PDe	PE	Means	SDs
LE	**0.826**	[0.674–0.774]	[0.667–0.767]	[0.624–0.736]	[0.527–0.667]	[0.609–0.733]	[0.566–0.701]	[0.398–0.568]	4.110	0.972
TP	0.724	**0.742**	[0.701–0.798]	[0.622–0.734]	[0.509–0.665]	[0.621–0.733]	[0.469–0.611]	[0.601–0.730]	4.106	0.769
SP	0.717	0.751	**0.734**	[0.575–0.715]	[0.482–0.642]	[0.521–0.671]	[0.479–0.639]	[0.519–0.669]	4.142	0.830
CP	0.680	0.679	0.645	**0.768**	[0.406–0.586]	[0.590–0.722]	[0.495–0.651]	[0.530–0.681]	4.264	0.875
PD	0.597	0.588	0.562	0.498	**0.734**	[0.425–0.585]	[0.285–0.455]	[0.321–0.510]	4.032	0.828
PI	0.671	0.678	0.596	0.658	0.505	**0.710**	[0.365–0.535]	[0.655–0.767]	4.938	0.893
PDe	0.634	0.541	0.559	0.575	0.362	0.449	**0.732**	[0.355–0.525]	4.457	0.918
PE	0.483	0.666	0.594	0.607	0.404	0.711	0.440	**0.716**	4.999	1.070

Diagonal entries are the square roots of AVE; entries below the diagonal are latent variable correlations from the final PLS model; entries above the diagonal are 95% bootstrap percentile confidence intervals for the latent variable correlations (5,000 resamples, two-tailed); LE (Learning Engagement), TP (Teaching Presence), SP (Social Presence), CP (Cognitive Presence), PD (Problem Driven), PI (Project Implementation), PDe (Project Design), PE (Project Evaluation). Bold diagonal values represent the square roots of the average variance extracted (AVE).

**Table 4:** Results of model path testing.

Model path	*β*	*p*-values	*T*	*f* ^2^	VIF	HTMT ratio	Test results
PD → LE	0.138	<0.001	4.144	0.042	1.672	0.535	Supported
PDe → LE	0.237	<0.001	7.290	0.123	1.663	0.575	Supported
PI→LE	0.305	<0.001	7.229	0.129	2.642	0.662	Supported
PE → LE	−0.255	<0.001	6.241	0.098	2.408	0.457	Supported
PD → CP	0.158	<0.001	4.266	0.041	1.387	0.524	Supported
PDe → CP	0.294	<0.001	8.933	0.150	1.337	0.548	Supported
PI→CP	0.307	<0.001	6.366	0.093	2.338	0.698	Supported
PE → CP	0.195	<0.001	3.963	0.042	2.105	0.564	Supported
PD → SP	0.284	<0.001	7.583	0.131	1.387	0.619	Supported
PDe → SP	0.279	<0.001	8.910	0.131	1.337	0.585	Supported
PI→SP	0.150	<0.001	3.020	0.021	2.338	0.660	Supported
PE → SP	0.249	<0.001	5.622	0.066	2.105	0.647	Supported
PD → TP	0.275	<0.001	9.588	0.147	1.387	0.564	Supported
PDe → TP	0.205	<0.001	7.254	0.085	1.337	0.547	Supported
PI→TP	0.236	<0.001	5.735	0.065	2.338	0.706	Supported
PE → TP	0.297	<0.001	7.693	0.113	2.105	0.586	Supported
TP → LE	0.213	<0.001	5.267	0.050	3.317	0.742	Supported
CP → LE	0.137	<0.001	4.019	0.027	2.481	0.704	Supported
SP → LE	0.228	<0.001	6.546	0.069	2.733	0.768	Supported

LE (Learning Engagement), TP (Teaching Presence), SP (Social Presence), CP (Cognitive Presence), PD (Problem Driven), PI (Project Implementation), PDe (Project Design), PE (Project Evaluation).

#### 4.1.3 Structural model assessment and hypothesis testing

We used Bootstrapping with 5,000 resamples to assess the significance and robustness of the structural paths. Table 4 reports the path analysis results.

#### 4.1.4 Net direct statistical associations of PBL dimensions with learning engagement

Problem Driven had a significant positive effect on Learning Engagement (*β* = 0.138, *t* = 4.144, *p* < 0.001), supporting H1a. Project Design also had a significant positive effect on Learning Engagement (*β* = 0.237, *t* = 7.290, *p* < 0.001), supporting H1b, as did Project Implementation (*β* = 0.305, *t* = 7.229, *p* < 0.001), supporting H1c. By contrast, Project Evaluation had a significant negative direct effect on Learning Engagement (*β* = −0.255, *t* = 6.241, *p* < 0.001). This result is consistent with the direction predicted by H1d and partially consistent with the previously reported nonsignificant direct effect of evaluation (Zhang and Lin, 2026). However, the present study shows that its direction depends on model specification and may reflect statistical suppression.

To assess the stability of the negative direct path from PE to LE, we compared four alternative models. In M1, which included PE as the sole predictor of LE, the PE path coefficient was positive (*β* = 0.483, *p* < 0.001), consistent with the positive correlation between PE and LE in Table 3 (*r* = 0.483). In M2, which included PD, PDe, PI, and PE simultaneously without mediators, the PE coefficient became negative (*β* = −0.131, *p* < 0.01). In M3, which excluded PI from the model without mediators, the PE coefficient remained negative (*β* = −0.067, *p* < 0.01). M4 was the full mediation model: the direct PE–LE coefficient was negative (*β* = −0.255, *p* < 0.001), and the total effect was *β* = −0.113 (95% CI [−0.197, −0.031], *p* < 0.01). The VIF values for PE and PI were below the conventional threshold of 5 (Table 4), ruling out severe multicollinearity. Nevertheless, the change from a positive coefficient in M1 to negative coefficients in M2 and M3 indicates sensitivity to model specification. This sign change suggests possible statistical suppression. Accordingly, throughout this article, the negative PE coefficient is interpreted only as a “net direct association after controlling for the other PBL dimensions and CoI mediators,” rather than as evidence that PE inhibits engagement when operating alone or in the absence of CoI support. The positive coefficient for PE as the sole predictor of LE in M1 must be taken into account before interpreting its negative net direct coefficient.

#### 4.1.5 Effects of PBL dimensions on the three CoI dimensions

Problem Driven had significant positive effects on Teaching Presence (*β* = 0.275, *t* = 9.588, *p* < 0.001), Social Presence (*β* = 0.284, t = 7.583, *p* < 0.001), and Cognitive Presence (*β* = 0.158, *t* = 4.266, *p* < 0.001). Project Design had significant positive effects on Teaching Presence (*β* = 0.205, *t* = 7.254, *p* < 0.001), Social Presence (*β* = 0.279, *t* = 8.910, *p* < 0.001), and Cognitive Presence (*β* = 0.294, t = 8.933, *p* < 0.001). Project Implementation had significant positive effects on Teaching Presence (β = 0.236, *t* = 5.735, *p* < 0.001), Social Presence (*β* = 0.150, *t* = 3.020, *p* < 0.001), and Cognitive Presence (*β* = 0.307, *t* = 6.366, *p* < 0.001). Project Evaluation had significant positive effects on Teaching Presence (*β* = 0.297, *t* = 7.693, *p* < 0.001), Social Presence (*β* = 0.249, *t* = 5.622, *p* < 0.001), and Cognitive Presence (*β* = 0.195, t = 3.963, *p* < 0.001).

#### 4.1.6 Effects of the three CoI dimensions on learning engagement

Teaching Presence (*β* = 0.213, *t* = 5.267, *p* < 0.001), Social Presence (*β* = 0.228, *t* = 6.546, *p* < 0.001), and Cognitive Presence (*β* = 0.137, *t* = 4.019, *p* < 0.001) all had significant positive effects on Learning Engagement, providing a basis for testing the mediating effects.

The model explained 52.4% of the variance in Learning Engagement (*R*^2^ = 0.524; adjusted *R*^2^ = 0.517), indicating moderately strong explanatory power (Dash and Paul, 2021). Explanatory power was also satisfactory for Teaching Presence (*R*^2^ = 0.371; adjusted *R*^2^ = 0.366), Cognitive Presence (*R*^2^ = 0.362; adjusted *R*^2^ = 0.357), and Social Presence (*R*^2^ = 0.416; adjusted *R*^2^ = 0.411). All endogenous latent variables had positive Stone-Geisser Q^2^ values. *Q*^2^ predict was 0.350 for Learning Engagement, 0.321 for Teaching Presence, 0.376 for Social Presence, and 0.297 for Cognitive Presence. Values above zero indicate predictive relevance. All *Q*^2^ values fell between 0.25 and 0.50, suggesting moderate predictive ability for the endogenous constructs (Dong et al., 2026). Taken together, the R^2^ and Q^2^ results indicate satisfactory explanatory power and predictive relevance, supporting the theoretical model’s validity and potential generalizability.

Effect sizes for the predictors of Learning Engagement ranged from 0.027 to 0.129. Project Implementation had the largest effect size (*f*^2^ = 0.129), interpreted as moderate, followed by Project Design (*f*^2^ = 0.123) and Project Evaluation (*f*^2^ = 0.098). Problem Driven had a smaller effect size (*f*^2^ = 0.042). Among the CoI dimensions, Social Presence (*f*^2^ = 0.069) and Teaching Presence (*f*^2^ = 0.050) had larger effect sizes than Cognitive Presence (*f*^2^ = 0.027). Effect sizes for predictors of the three CoI dimensions ranged from 0.021 to 0.150; the effects of Project Design on Cognitive Presence (*f*^2^ = 0.150) and Project Evaluation on Teaching Presence (*f*^2^ = 0.113) were interpreted as moderate. Variance inflation factors (VIF) ranged from 1.337 to 3.317, all below the threshold of 5.000 (Diamantopoulos and Siguaw, 2006), indicating no severe multicollinearity. All HTMT ratios were below the conservative threshold of 0.85 (Henseler et al., 2015), indicating no serious discriminant validity problems under the HTMT criterion. However, the Fornell-Larcker criterion was not met for TP and SP; therefore, the evidence provides partial support for discriminant validity rather than fully establishing it. Paths involving TP and SP require cautious estimation and interpretation.

#### 4.1.7 Mediating effect tests

To test whether the three CoI dimensions mediate the relationships between PBL dimensions and Learning Engagement, we used Bootstrapping with 5,000 resamples and two-tailed tests to estimate indirect effects and their confidence intervals. Table 5 presents the results.

**Table 5:** Results of Tests of Indirect Statistical Associations.

Path	*β*	SD	*T*	*p*	CI
PD → TP → LE	0.056	0.012	4.599	<0.001	[0.032, 0.080]
PD → CP → LE	0.017	0.007	2.608	0.009	[0.006, 0.032]
PD → SP → LE	0.060	0.013	4.560	<0.001	[0.036, 0.087]
PI→TP → LE	0.046	0.013	3.577	<0.001	[0.023, 0.073]
PI→CP → LE	0.034	0.012	2.893	0.004	[0.013, 0.059]
PI→SP → LE	0.030	0.012	2.585	0.010	[0.010, 0.056]
PDe → TP → LE	0.042	0.010	4.180	<0.001	[0.023, 0.063]
PDe → CP → LE	0.031	0.010	3.139	0.002	[0.012, 0.051]
PDe → SP → LE	0.058	0.011	5.113	<0.001	[0.037, 0.082]
PE → TP → LE	0.065	0.016	4.112	<0.001	[0.036, 0.098]
PE → CP → LE	0.023	0.009	2.479	0.013	[0.008, 0.044]
PE → SP → LE	0.054	0.013	4.038	<0.001	[0.031, 0.084]

Problem Driven had significant indirect statistical associations with Learning Engagement through Teaching Presence (*β* = 0.056, 95% CI [0.032, 0.080], *p* < 0.001), Cognitive Presence (*β* = 0.017, 95% CI [0.006, 0.032], *p* = 0.009), and Social Presence (*β* = 0.060, 95% CI [0.036, 0.087], *p* < 0.001). None of the three confidence intervals included zero, indicating significant mediating effects and supporting H2a, H2b, and H2c.

Project Implementation had significant indirect statistical associations with Learning Engagement through Teaching Presence (*β* = 0.046, 95% CI [0.023, 0.073], *p* < 0.001), Cognitive Presence (*β* = 0.034, 95% CI [0.013, 0.059], *p* = 0.004), and Social Presence (*β* = 0.030, 95% CI [0.010, 0.056], *p* = 0.010). None of the three confidence intervals included zero, indicating significant mediating effects and supporting H2a, H2b, and H2c.

Project Design had significant indirect statistical associations with Learning Engagement through Teaching Presence (β = 0.042, 95% CI [0.023, 0.063], *p* < 0.001), Cognitive Presence (*β* = 0.031, 95% CI [0.012, 0.051], *p* = 0.002), and Social Presence (*β* = 0.058, 95% CI [0.037, 0.082], *p* < 0.001). None of the three confidence intervals included zero, indicating significant mediating effects and supporting H2a, H2b, and H2c.

Project Evaluation had significant indirect statistical associations with Learning Engagement through Teaching Presence (*β* = 0.065, 95% CI [0.036, 0.098], *p* < 0.001), Cognitive Presence (*β* = 0.023, 95% CI [0.008, 0.044], *p* = 0.013), and Social Presence (*β* = 0.054, 95% CI [0.031, 0.084], *p* < 0.001). None of the three confidence intervals included zero, indicating significant mediating effects and supporting H2a, H2b, and H2c.

Of particular note, after controlling for the other PBL dimensions and CoI mediators, the net direct path coefficient from Project Evaluation to Learning Engagement was negative, and the total association was *β* = −0.113. The three indirect paths summed to *β* = 0.142, offsetting approximately 55.7% of the negative net direct path coefficient (0.142/0.255), but did not reverse the direction of the total association after controlling for the other variables. This indicates that PE’s negative net direct association and positive indirect associations coexist, with the latter providing only a partial offset rather than a complete reversal. It should be emphasized that PE had a positive zero-order association with LE, and its coefficient was also positive when it alone predicted LE in M1. Thus, the negative net direct coefficient cannot be interpreted as evidence that evaluation inhibits engagement when it “operates alone” or “lacks CoI support.” A more cautious interpretation is that, when PE’s unique variance is separated from the other PBL dimensions and CoI experiences, its net direct statistical association is negative, possibly reflecting statistical suppression, whereas its indirect statistical associations through the activation of CP, SP, and TP are positive and may partially offset this negative net direct association. The central question is not “whether evaluation itself is effective,” but “under which model specifications and combinations of conditions evaluation exhibits a negative net direct association, and how CoI structures may offset this net association.”

Overall, Teaching Presence showed the highest indirect statistical associations across the paths involving the PBL dimensions (indirect statistical association *β* = 0.042–0.065), followed by Social Presence (*β* = 0.030–0.060), with comparatively weaker associations for Cognitive Presence (*β* = 0.017–0.034). Because differences between indirect effects were not formally tested, we do not claim that any CoI dimension has the “most robust” or “most critical” indirect association.

### 4.2 Study 2 fuzzy set qualitative comparative analysis (fsQCA)

#### 4.2.1 Data calibration

Calibration converts raw scale scores into fuzzy-set membership scores ranging from 0 to 1, representing the degree to which each case belongs to a given set. The analysis included seven antecedent conditions—Project Evaluation, Project Implementation, Project Design, Problem Driven, Cognitive Presence, Social Presence, and Teaching Presence—with Learning Engagement as the outcome.

We used direct calibration (Rihoux, 2006), specifying three anchors corresponding to full membership (0.95), the crossover point (0.50), and full non-membership (0.05). To avoid observations coinciding exactly with the calibration anchors and being lost through boundary effects, each anchor was increased by 0.001 while preserving the distances between anchors, thereby retaining all cases (Fiss, 2011). Table 6 reports the calibration anchors for each variable.

**Table 6:** Calibration anchors for fsQCA.

Variable	Full membership (0.95)		Crossover point (0.50)		Full nonmembership (0.05)	
	Original	Modified	Original	Modified	Original	Modified
Problem Driven	5.000	5.001	4.000	4.001	3.058	3.059
Project Design	5.592	5.593	4.500	4.501	3.333	3.334
Project Evaluation	6.364	6.365	5.000	5.001	3.714	3.715
Project Implementation	6.000	6.001	5.000	5.001	3.833	3.834
Cognitive Presence	5.400	5.401	4.200	4.201	3.200	3.201
Social Presence	5.200	5.201	4.000	4.001	3.200	3.201
Teaching Presence	5.000	5.001	4.200	4.201	3.200	3.201
Learning Engagement	5.333	5.334	4.111	4.112	2.778	2.779

#### 4.2.2 Necessary condition analysis

Necessity analysis examines whether a condition must be present for high Learning Engagement to occur. Following Fiss (2011), a condition is considered necessary when its Consistency reaches or exceeds 0.90. As shown in Table 7, consistency values for all conditions and their negations (~) were below this threshold. Social Presence had the highest consistency (0.830), followed by Cognitive Presence (0.791), Project Implementation (0.761), and Problem Driven (0.760), but none met the criterion for necessity. Thus, no individual PBL or CoI dimension was indispensable for high Learning Engagement. This finding complements the PLS-SEM results by suggesting that engagement reflects the interplay of multiple factors rather than a single dominant condition, providing a basis for the subsequent analysis of configurational sufficiency.

**Table 7:** Necessary condition analysis.

Condition	Consistency	Coverage
Problem Driven	0.760	0.774
~Problem Driven	0.541	0.561
Project Design	0.753	0.798
~Project Design	0.563	0.561
Project Evaluation	0.728	0.756
~Project Evaluation	0.586	0.596
Project Implementation	0.761	0.811
~Project Implementation	0.573	0.568
Cognitive Presence	0.791	0.802
~Cognitive Presence	0.525	0.546
Social Presence	0.830	0.803
~Social Presence	0.498	0.546
Teaching Presence	0.758	0.854
~Teaching Presence	0.566	0.535

#### 4.2.3 Configurational sufficiency analysis

Sufficiency analysis identifies combinations of conditions associated with high Learning Engagement. With seven antecedent conditions (Project Evaluation, Project Implementation, Project Design, Problem Driven, Cognitive Presence, Social Presence, and Teaching Presence), the truth table comprised 2^7^ = 128 logically possible combinations. Following Fiss (2011), we set the consistency threshold at 0.80 and the case frequency threshold at 3 to ensure the empirical relevance of the configurations.

Boolean minimization was conducted in fsQCA 3.0 to obtain complex, intermediate, and parsimonious solutions. The complex solution used only observed configurations, without counterfactual assumptions, and comprised nine configurations (PDe*PD*CP*SP; PI*PDe*CP*TP; PDe*PD*SP*TP; PE*PDe*PD*CP*TP; PE*PDe*CP*SP*TP; PE*PD*CP*SP*TP; PI*PD*CP*SP*TP; ~PE*PI* ~ PDe* ~ PD* ~ CP* ~ SP* ~ TP; ~PE* ~ PI* ~ PDe* ~ PD* ~ CP*SP* ~ TP), with overall coverage of 0.741 and consistency of 0.857. The parsimonious solution allowed all counterfactual combinations and comprised four configurations: PE*PDe*TP; PDe*PD*TP; PE*PD*CP*SP; PI*PD*CP*SP. Its overall coverage was 0.713 and consistency was 0.899. This solution was used to identify core conditions. The intermediate solution allowed only easy counterfactuals consistent with theoretically informed directional expectations, balancing parsimony and theoretical interpretability. For high LE, the presence of PD, PDe, PI, CP, SP, and TP was expected. No directional expectation was specified for PE because its direct association was non-positive whereas its indirect statistical associations were positive; PE was therefore allowed to enter the intermediate solution without a directional restriction. Conditions appearing in both the intermediate and parsimonious solutions were classified as core, whereas those appearing only in the intermediate solution were classified as peripheral. Table 8 reports the intermediate solution, which comprised seven sufficient configurations. Overall solution consistency was 0.924, well above the acceptable threshold of 0.80, and overall coverage was 0.667, indicating that the seven configurations jointly accounted for 66.7% of high Learning Engagement cases and provided substantial explanatory coverage.

**Table 8:** High levels of learning engagement.

Construct	Learning Engagement						
	C1	C2	C3	C4	C5	C6	C7
Problem Driven (PD)	●	●	•		●	●	●
Project Design (PDe)	●	•	●	●			●
Project Evaluation (PE)		●	●	●	●		•
Project Implementation (PI)	Ⓧ					●	•
Cognitive Presence (CP)		●		•	●	●	•
Social Presence (SP)	•	●	•	•	●	●	
Teaching Presence (TP)	●		●	●	•	•	●
Consistency	0.964	0.947	0.951	0.958	0.951	0.957	0.962
Raw coverage	0.345	0.447	0.438	0.474	0.475	0.490	0.302
Unique coverage	0.023	0.031	0.006	0.012	0.009	0.024	0.012
Solution consistency	0.924						
Solution coverage	0.667						

C, Configuration; ● indicates the presence of a core condition; • indicates the presence of a peripheral condition; Ⓧ indicates the absence (negation) of a core condition. A blank cell denotes a “do not care” condition: its presence or absence does not alter the sufficiency of the corresponding configuration for high Learning Engagement.

Configuration C1 (PD*PDe* ~ PI*SP*TP) comprises Problem Driven, Project Design, and Teaching Presence as core conditions, Social Presence as a peripheral condition, and the explicit negation of Project Implementation (~PI). Project Evaluation and Cognitive Presence are “do not care” conditions. Its consistency was 0.964, the highest among the seven configurations, and its raw coverage was 0.345. The term ~PI is the only explicitly negated condition in the seven-configuration solution. It indicates that the absence of Project Implementation, together with the other specified conditions, is sufficient for high engagement. Blank cells elsewhere do not indicate absence. This configuration suggests that strong design, problem orientation, and instructional support can compensate for weaknesses in Project Implementation.

Configuration C2 (PD*PDe*PE*CP*SP) comprises Problem Driven, Project Evaluation, Cognitive Presence, and Social Presence as core conditions, with Project Design as a peripheral condition. Project Implementation and Teaching Presence are “do not care” conditions. Its consistency was 0.947 and its raw coverage was 0.447. This configuration indicates that high set membership in Problem Driven, Project Evaluation, Cognitive Presence, and Social Presence, together with peripheral support from Project Design, can constitute a sufficient configuration for high Learning Engagement.

Configuration C3 (PD*PDe*PE*SP*TP) comprises Project Design, Project Evaluation, and Teaching Presence as core conditions, with Problem Driven and Social Presence as peripheral conditions. Project Implementation and Cognitive Presence are “do not care” conditions. Its consistency was 0.951, raw coverage was 0.438, and unique coverage was 0.006. This pathway suggests that when Project Implementation and Cognitive Presence are unspecified, the core combination of PBL evaluation and design with Teaching Presence, supported peripherally by Problem Driven and Social Presence, can still be associated with high Learning Engagement. The low unique coverage (0.6%) indicates that most cases covered by this pathway were also covered by other configurations.

Configuration C4 (PDe*PE*CP*SP*TP) comprises Project Design, Project Evaluation, and Teaching Presence as core conditions, with Cognitive Presence and Social Presence as peripheral conditions. Its consistency was 0.958, raw coverage was 0.474, and unique coverage was 0.012. Even when Problem Driven and Project Implementation are unspecified, high Learning Engagement can be achieved when Project Evaluation and Project Design are present as core design conditions and the cognitive, social, and teaching dimensions of CoI work together as core or peripheral conditions. This pathway particularly highlights the central contribution of Project Evaluation when embedded in a strong design framework and supported by CoI experiences.

Configuration C5 (PD*PE*CP*SP*TP) comprises Problem Driven, Project Evaluation, Cognitive Presence, and Social Presence as core conditions, with Teaching Presence as a peripheral condition. Project Design and Project Implementation are “do not care” conditions. Its consistency was 0.951, raw coverage was 0.475, and unique coverage was 0.009. When the design and implementation dimensions of PBL are unspecified, the core combination of Project Evaluation and Problem Driven with Cognitive Presence and Social Presence, accompanied by peripheral Teaching Presence, is sufficient for high Learning Engagement. Although Teaching Presence is peripheral in this configuration, its supporting role remains integral to the specified combination.

Configuration C6 (PD*PI*CP*SP*TP) comprises Problem Driven, Project Implementation, Cognitive Presence, and Social Presence as core conditions, with Teaching Presence as a peripheral condition. Project Design and Project Evaluation are “do not care” conditions. Its consistency was 0.957, raw coverage was 0.490, the highest among the seven configurations, and unique coverage was 0.024. When the upstream design elements of PBL (evaluation and design) are unspecified, authentic Project Implementation and Problem Driven, combined with core Cognitive Presence and Social Presence and peripheral Teaching Presence, are sufficient for high engagement.

Configuration C7 (PD*PDe*PE*PI*CP*TP) comprises Problem Driven, Project Design, and Teaching Presence as core conditions, with Project Evaluation, Project Implementation, and Cognitive Presence as peripheral conditions. Social Presence is a “do not care” condition. Its consistency was 0.962, raw coverage was 0.302, the lowest among the seven configurations, and unique coverage was 0.012. When Social Presence is unspecified, the combination of all four PBL dimensions with Teaching Presence and Cognitive Presence can still be associated with high engagement. Its comparatively low raw coverage indicates that this pathway applies to a smaller share of high-engagement cases.

#### 4.2.4 Key findings

First, no single PBL dimension was necessary across all seven configurations. Each configuration included at least one CoI dimension as a core or peripheral condition; C4, C5, and C6 included all three. However, fsQCA identifies sufficient configurations for high Learning Engagement and does not test indirect or mediating effects. These findings therefore do not test the indirect statistical associations estimated by PLS-SEM. They provide complementary configurational evidence that CoI-related experiences are common constituent conditions of high-engagement configurations.

Project Evaluation exhibits a pattern of conditional presence in high-engagement configurations. In PLS-SEM, PE had a negative net direct association after controlling for the other variables (*β* = −0.255). In fsQCA, PE appeared as a core or peripheral condition in five configurations (C2, C3, C4, C5, and C7), and as a “do not care” condition in C1 and C6. This distribution only indicates that PE can combine with different conditions to constitute sufficient configurations for high LE; it does not test whether the strength of the statistical association between PE and LE varies with CoI levels.

#### 4.2.5 Robustness checks

Following Schneider and Wagemann (2010), we assessed predictive validity by randomly dividing the full sample into training (*n* = 245) and test (*n* = 245) sets. Re-estimating fsQCA in the training set yielded a configurational structure closely aligned with the full-sample solution (overall consistency = 0.908; overall coverage = 0.627). Applying the training-set configuration rules to the test set produced predictive consistency of 0.897 and predictive coverage of 0.541. Both exceeded the recommended thresholds (consistency >0.80; coverage >0.50), supporting predictive robustness. We also varied the consistency threshold from 0.8 to 0.85 and the case frequency threshold from 2 to 5. The overall configurational structure and core conditions remained stable, and coverage changed by less than 7%, indicating limited sensitivity to these parameter choices (Schneider and Wagemann, 2010).

## 5 Discussion and implications

### 5.1 Theoretical contributions

First, this study identifies a negative net direct association involving Project Evaluation after controlling for the other PBL dimensions and CoI mediators, as well as the conditional presence of PE in high-engagement configurations. Prior research described “ineffective or negative evaluation effects” as a static finding (Zhang and Lin, 2026). The present study, however, shows that PE had a positive zero-order association with LE and a positive coefficient when used as the sole predictor. The negative coefficient emerged only after controlling for the other PBL dimensions and CoI mediators and was sensitive to model specification, suggesting possible statistical suppression. Thus, this negative association should be restricted to a “net direct statistical association” and cannot be equated with the effect of evaluation itself in isolation. In PLS-SEM, PE’s indirect statistical associations through the three CoI dimensions were positive and partially offset the negative net direct association in the total effect, but did not reverse the direction of the total association after controlling for the other variables. It must be made clear that fsQCA does not test moderation or mediation and therefore cannot demonstrate that CoI statistically buffers PE’s negative coefficient. The fsQCA results only show that PE appeared as a core or peripheral condition in five high-engagement configurations and as a “do not care” condition in C1 and C6, indicating that PE can combine with different conditions to constitute sufficient configurations for high LE. PLS-SEM and fsQCA have different analytical logics: the former estimates net direct and indirect statistical associations, whereas the latter identifies sufficient configurations for high engagement. They therefore provide complementary evidence rather than corroborating each other. Taken together, evaluation is neither inherently facilitative nor inherently inhibitory: its zero-order association is positive, its net direct statistical association after controlling for the other variables is negative, and its presence in high-engagement configurations is conditional.

Second, this study establishes CoI as a structural framework for examining indirect statistical associations and configurational conditions linking PBL design to Learning Engagement. All 12 indirect paths from the PBL dimensions through the three CoI dimensions to Learning Engagement were significant. The point estimates of the indirect statistical associations through Teaching Presence and Social Presence were relatively higher, but their relative magnitudes were not consistent across the PBL dimensions. Because differences between indirect effects were not formally tested, we do not claim that any dimension has the strongest statistical mediation association. This contribution extends beyond re-examining the applicability of CoI to technology-enhanced learning, for which prior research has already provided initial evidence (Yulian et al., 2025). By repositioning CoI as a framework of statistical mediators and configurational conditions, we clarify how perceptions of instructional design and experiences within the learning environment are jointly associated with individual engagement. This perspective helps explain why the same PBL design element may exhibit heterogeneous associations under different levels of reported CoI experiences. The fsQCA results further show that every high-engagement configuration contains at least one CoI dimension as a core or peripheral condition, although only C4, C5, and C6 include CP, SP, and TP together. These findings complement the indirect statistical associations from PLS-SEM, but the methods have distinct analytical logics: PLS-SEM estimates average indirect statistical associations, whereas fsQCA identifies sufficient configurations for high engagement. Accordingly, fsQCA cannot test mediating effects or confirm a generative mechanism involving CoI. We interpret its results only as complementary evidence that CoI dimensions consistently feature in high-engagement configurations.

Third, fsQCA reveals multiple equifinal pathways to high Learning Engagement, demonstrating that individual PBL dimensions are not universally required and that CoI dimensions can combine flexibly. No single PBL dimension was necessary across all seven configurations. Project Implementation was absent in C1, Problem Driven was a “do not care” condition in C4, and Project Design was a “do not care” condition in C5, yet each configuration was associated with high engagement. This distribution suggests that a weaker contribution from one PBL dimension can be compensated for by other PBL dimensions and CoI conditions. Project Evaluation appeared as a core or peripheral condition in five configurations, despite its negative direct path coefficient in PLS-SEM. A positive evaluation–engagement association may be more likely when evaluation co-occurs with CoI conditions, as illustrated by C2, C4, and C5. This configurational evidence extends the interpretation of Project Evaluation beyond a net-effect perspective: its association with engagement depends on the combination of conditions in which it is embedded.

Fourth, integrating PLS-SEM and fsQCA offers PBL research an analytical approach that captures both net-effect associations and equifinal configurational pathways. Educational technology research predominantly uses symmetric methods, such as regression and SEM, to estimate average net effects (Alkawsi et al., 2025). These methods assume symmetry and additivity in associations and are less suited to identifying equifinal pathways. In this study, the PLS-SEM findings (12 significant indirect paths) characterize average indirect statistical associations, whereas the fsQCA findings (seven equifinal configurations) identify multiple sufficient combinations associated with high engagement. The methodological contribution lies in enabling researchers to select net-effect or configurational analysis according to their research questions, or to use both as complementary sources of evidence.

### 5.2 Practical implications

The findings inform the following recommendations for PBL design in animation education.

Resource allocation can be differentiated to take advantage of flexibility across CoI configurations. The fsQCA findings indicate that high Learning Engagement does not require all CoI dimensions to be simultaneously high or all PBL dimensions to be jointly present. C1 includes the explicit negation of Project Implementation (~PI), suggesting that stronger Problem Driven, Project Design, and Teaching Presence may compensate when implementation is weak. C5 indicates that high engagement remains possible when Project Design and Project Implementation are unspecified, provided that Problem Driven, Project Evaluation, and all three CoI dimensions co-occur. Resource-constrained programs can therefore prioritize particular CoI dimensions strategically. Courses with limited instructor capacity might invest in peer collaboration infrastructure, including structured peer review and team project milestones, to strengthen Social Presence. Independent creative courses offering fewer opportunities for peer interaction might prioritize scaffolds for Teaching Presence, such as instructor-led visual argumentation meetings. Practitioners should select feasible pathways for activating CoI based on their available resources rather than attempting to maximize all three dimensions equally.

### 5.3 Limitations and future research

This study has several limitations. First, the sample was restricted to upper-year animation students at application-oriented undergraduate institutions in eastern China. Although this choice was theoretically motivated, generalization to other disciplines, institution types, and educational levels requires caution. Second, the temporal design constrains interpretation. Learning Engagement was measured at T2, but all PBL and CoI dimensions were measured concurrently at T1. The findings therefore support indirect statistical associations in which T1 perceptions of PBL and CoI are jointly associated with T2 Learning Engagement; they do not establish the temporal sequence PBL → CoI → Engagement. Reverse associations cannot be fully excluded: for example, CoI experiences may shape students’ retrospective perceptions of PBL design. Common method bias within T1, third variables, and course-level contextual factors may also affect path estimates. Future research should use three-wave longitudinal designs measuring PBL at T1, CoI at T2, and LE at T3. Cross-lagged panel models or random-intercept cross-lagged panel models could provide more rigorous tests of temporal mediation.

Third, the empirical role of GenAI is limited. Although the study was conducted in AIGC-assisted PBL studios and all participants attended courses using AIGC tools, we did not measure AI use in terms of type, purpose, frequency, intensity, quality, dependence, or AI literacy, nor did we include a comparison group with no or low AI use. GenAI is therefore a contextual feature and an eligibility criterion, rather than an explanatory variable or an empirically examined correlate. The findings cannot be attributed to GenAI itself and apply only to comparable AIGC-assisted PBL settings. Future studies should develop or adopt measures of AI use and incorporate its intensity, type, quality, dependence, and AI literacy into the model. Comparisons with no- or low-AI-use groups, multigroup analysis, longitudinal designs, or experiments could then examine the role of GenAI in the PBL–CoI–Learning Engagement relationship.

Fourth, the evidence for the discriminant validity of TP and SP is mixed. Although all HTMT values were below the conservative threshold of 0.85, the Fornell-Larcker criterion was not met: the correlation between TP and SP was 0.751, exceeding the square roots of AVE for TP (0.742) and SP (0.734). This indicates that the two constructs are highly correlated at the measurement level. Future research could further improve the distinctiveness of TP and SP items or test a higher-order factor structure for CoI to establish their construct boundaries more rigorously. Accordingly, paths involving TP and SP are interpreted cautiously in this article, and discriminant validity is not considered fully established.

Fifth, the measurement structure of Learning Engagement presents a limitation. This study modeled the nine LE items as direct indicators of a single reflective construct. Behavioral, emotional, and cognitive engagement were not specified as three first-order factors, and a higher-order LE construct was not tested. Therefore, the conclusions concern overall learning engagement and do not support inferences about the effect patterns of the three individual dimensions. Future research could test a three-dimensional measurement structure, a higher-order factor structure, or dimension-specific paths for LE to characterize the different aspects of learning engagement more precisely.

## 6 Conclusion

High Learning Engagement is not necessarily determined by any single PBL dimension, but is associated with the joint presence of multiple PBL dimensions and the three CoI dimensions in particular configurations. This pattern is characterized by multiple sufficient combinations and the absence of any universally required individual dimension. The direction of the association involving Project Evaluation depends on the conditions with which it co-occurs.

The conditional distribution of Project Evaluation in fsQCA has theoretical implications for its significant negative net direct path coefficient in PLS-SEM (*β* = −0.255). Evaluation appeared as a core or peripheral condition in five high-engagement configurations (C2, C3, C4, C5, and C7), and as a “do not care” condition in C1 and C6 rather than as an explicitly negated condition. This configurational distribution indicates that evaluation is not a necessary condition for high engagement, but can combine with different conditions to constitute sufficient configurations for high engagement. It should be emphasized that fsQCA does not test moderation or mediation; therefore, these results cannot be interpreted as evidence that CoI statistically buffers PE’s negative coefficient. PLS-SEM and fsQCA provide evidence of net direct associations and configurational sufficiency, respectively. They have different analytical logics and cannot substitute for each other.

In PLS-SEM, PE’s negative net direct path coefficient emerged only after controlling for the other PBL dimensions and CoI mediators. In the baseline model M1, which included only PE and LE, the path coefficient for PE as the sole predictor of LE was positive, consistent with the positive zero-order correlation. Therefore, the negative coefficient cannot be interpreted as evidence that evaluation itself inhibits learning engagement or does so “in the absence of CoI support.” It represents only a net direct statistical association that depends on a particular model specification and may arise from statistical suppression. PE’s positive indirect statistical associations through CoI partially offset this negative net direct association in the total effect, but did not reverse the direction of the total association after controlling for the other variables. This result supplements and refines a singular interpretation of evaluation’s net effect in conventional linear models, indicating that its net direct association and its presence in configurations are conditional. It does not demonstrate that CoI statistically moderates or buffers PE’s negative coefficient. In practice, PE’s positive zero-order association indicates that the value of evaluation itself should not be dismissed. A more robust design principle is to integrate evaluation into a CoI support system to reduce the possibility that its unique variance exhibits a negative net direct association after controlling for the other variables, rather than reducing evaluation to an isolated control mechanism.

Positioning CoI as both statistical mediators and configurational conditions has methodological implications. We used PLS-SEM to estimate indirect statistical associations between PBL inputs and engagement, and fsQCA to identify multiple sufficient configurations for high engagement. The methods provide complementary evidence from distinct perspectives: PLS-SEM characterizes average indirect associations, while fsQCA reveals how conditions combine within high-engagement configurations.

From a configurational perspective, this study systematically identifies multiple equifinal configurational pathways through which PBL design elements and CoI experiences are jointly associated with Learning Engagement. It clarifies the conditional boundaries of Project Evaluation effects and offers a theoretically differentiated and methodologically rigorous framework for understanding the complex relationships between instructional design and learning engagement. These conclusions remain specific to AIGC-assisted PBL studios and do not establish the instructional effects of generative AI itself.

## Data Availability

The raw data supporting the conclusions of this article will be made available by the authors, without undue reservation.
