# Supplementary material for: How does community of inquiry matter? Mediating effects and configurational pathways linking project-based learning design to learning engagement
Source: Front Psychol. 2026 Oct 23;17:1976588. doi: 10.3389/fpsyg.2026.1976588 (PMC13739778; doi:10.3389/fpsyg.2026.1976588)
Supplement: Supplementary file 1 [file Data_Sheet_1.docx]

Appendix A1. Questionnaire

**Background Information**

Gender: Male / Female

Year of study: Third year / Fourth year

Field ~~of study:~~

Institution type: Comprehensive university / Art and design institution

Did you participate in a PBL studio course supported by AIGC visual generation tools during the 2025–2026 academic year? Yes / No

During the past semester, how frequently did you use AIGC visual generation tools to support animation PBL projects?

Less than once per week / 1–2 times per week / 3 or more times per week

How long have you used AIGC tools to support animation creation?

Less than 6 months / 6–12 months / More than 12 months

I. PBL Scale

Dimension 1: Problem Driven

| PD1 | The central problem of the animation project is closely grounded in industry practice and offers substantial opportunities for visual inquiry. |
| --- | --- |
| PD2 | I enjoy solving authentic and interesting problems in animation creation. |
| PD3 | The way the animation project's problem is framed stimulates my curiosity and desire to explore visual solutions. |
| PD4 | The project's driving question prompts me to move beyond rapid AI outputs and question them critically. |
| PD5 | I enjoy discussing creative topics related to real needs in the animation industry. |
| PD6 | The project's central problem is sufficiently open-ended to allow multiple visual solutions. |

Dimension 2: Project Design

| PDs1 | The animation project's learning objectives and creative tasks are clearly structured. |
| --- | --- |
| PDs2 | The course's learning activities align with the goals of animation education. |
| PDs3 | Project Design provides adequate creative resources, including AI tools. |
| PDs4 | The project tasks match my abilities and are appropriately challenging. |
| PDs5 | Project Design gives me sufficient autonomy to explore visual possibilities. |
| PDs6 | Project Design effectively supports teamwork and interaction in the studio. |

Dimension 3: Project Implementation

| PI1 | During Project Implementation, I have sufficient opportunities for hands-on animation production. |
| --- | --- |
| PI2 | I can apply theoretical knowledge of animation to visual creative practice. |
| PI3 | During Project Implementation, my peers and I collaborate effectively. |
| PI4 | During animation creation, I receive timely guidance and feedback that help move the project forward. |
| PI5 | Studio teamwork strengthens my motivation to create. |
| PI6 | With AI assistance, I can generate and refine visual materials more efficiently. |

Dimension 4: Project Evaluation

| PE1 | Project Evaluation emphasizes reflection on the creative process rather than focusing solely on the final work. |
| --- | --- |
| PE2 | Evaluation activities, such as visual argumentation at project milestones, deepen my understanding of the project. |
| PE3 | Project Evaluation requires critical examination of AI-generated visual materials. |
| PE4 | Peer review strengthens my sense of involvement in the creative process. |
| PE5 | Instructor evaluation gives me clear directions for improvement. |
| PE6 | Project Evaluation encourages me to reflect on my own creative process. |
| PE7 | The evaluation criteria are clear and transparent and help me understand the creative goals. |

II. CoI Scale

Dimension 1: Cognitive Presence

| CP1 | AI-generated visual materials prompt me to reflect more deeply on design proposals. |
| --- | --- |
| CP2 | Through repeated visual argumentation during evaluation, I can develop and deepen my understanding of the work. |
| CP3 | The visual outputs of AI tools help me identify blind spots in my creative work and develop aesthetic judgment. |
| CP4 | In the PBL studio, I can construct and confirm the meaning of my creative work through sustained reflection and discourse. |
| CP5 | Critical discussions within the project deepen my understanding of animation as a discipline. |

Dimension 2: Social Presence

| SP1 | In the PBL studio, I develop close emotional connections with my peers. |
| --- | --- |
| SP2 | Shared AI-generated visual materials make team communication more efficient. |
| SP3 | I feel a strong sense of belonging to the team in the human–AI–human collaboration process. |
| SP4 | Peer review and team negotiation strengthen my creative motivation. |
| SP5 | In the PBL studio, I feel that I am a valued and trusted team member. |

Dimension 3: Teaching Presence

| TP1 | The instructor's clear guidance on the rules for using AI visual tools helps me define the direction of my creative work. |
| --- | --- |
| TP2 | Cognitive scaffolds designed by the instructor, such as visual argumentation templates, effectively support my deep learning. |
| TP3 | The instructor provides sufficient guidance when I critically select visual outputs generated by AI. |
| TP4 | The instructor's project design gives me a clear sense of direction and support for my creative work. |
| TP5 | Through evaluation and feedback at project milestones, the instructor makes me feel consistently supported and attended to. |

III. Learning Engagement Scale

Dimension 1: Behavioral Engagement

| BE1 | I am willing to invest more time than required in AI-assisted animation PBL projects. |
| --- | --- |
| BE2 | In the studio, I consistently remain focused and actively engaged in my work. |
| BE3 | I proactively seek additional creative resources, including AI tools, to complete project tasks. |

Dimension 2: Emotional Engagement

| EE1 | I feel excited and enthusiastic about AI-enhanced animation PBL courses. |
| --- | --- |
| EE2 | I experience a strong sense of achievement and satisfaction while learning in the studio. |
| EE3 | I am interested in learning and creating in the PBL studio. |

Dimension 3: Cognitive Engagement

| CE1 | Using AI tools prompts me to analyze visual solutions more deeply and exercise aesthetic judgment. |
| --- | --- |
| CE2 | In animation projects, I actively apply critical thinking to examine AI-generated visual solutions. |
| CE3 | I use a variety of creative strategies to solve problems encountered in animation projects. |
